# Assessment of adenosine triphosphate content in sausages stuffed in different modified casing treatments added with orange extracts, utilising hyperspectral imaging combined with multivariate analysis

**DOI:** 10.3389/fnut.2024.1370339

**Published:** 2024-03-04

**Authors:** Chao-Hui Feng, Hirofumi Arai, Francisco J. Rodríguez-Pulido

**Affiliations:** ^1^School of Regional Innovation and Social Design Engineering, Faculty of Engineering, Kitami Institute of Technology, Kitami, Hokkaido, Japan; ^2^RIKEN Centre for Advanced Photonics, RIKEN, Sendai, Miyagi, Japan; ^3^Food Colour and Quality Laboratory, Facultad de Farmacia, Universidad de Sevilla, Sevilla, Spain

**Keywords:** adenosine triphosphate, hyperspectral imaging, sausage, orange extracts, Kennard-stone algorithm

## Abstract

**Introduction:**

An investigation was conducted using a hyperspectral imaging (HSI) system to non-invasively estimate adenosine triphosphate (ATP) content in vacuum packaged sausages in different modified casing treatments added with orange extracts after a year of storage at 4°C.

**Methods:**

Various pre-processing combinations were applied to the spectra to enhance the performance of partial least squares regression (PLSR).

**Results and discussion:**

PLSR models, utilising the full absorbance spectrum with pre-treatment of standard normal variate combined with 1^st^ derivative,exhibited prediction coefficients of determination (R_p_^2^) reaching up to 0.6629. A distribution map developed through MATLAB was employed to display the location and concentration of ATP content in these unique sausages for the first time. The integration of HSI and multivariate analysis not only quantifies but also visually represents the changes in ATP content response to the different casing treatments, demonstrating the significant potential for real-time inspection in the processed meat industry.

## 1 Introduction

Citrus fruits, cultivated globally for commercial purposes, represent a crucial source for the fruit juice, jams, and jellies industry, play a significant role in job creation and offering substantial benefits (1, 2). Annually, the citrus-processing industry generates over forty million tons of waste (3). Notably, orange (*Citrus sinensis*) alone contributes over sixteen million tons of waste, comprising orange peel (60–65%) and pulp and seeds (30–35%) (4). In Japan, an estimated 400 thousand tons of fruit juice are consumed annually, with the JA (Japan Agricultural Cooperative) Foods Factory in Shizuoka prefecture processing around 2000 tons of oranges yearly, resulting in 1000 tons of waste (5). However, this waste incurs significant costs to follow Japan’s Food Recycling Law. Typically, waste orange peels are handled by direct landfill disposal as fertilizer, animal feed utilization, or sale as dried orange peels to China as it is one of the components in Chinese herbal medicine. Economically, recycling waste orange peels can address environmental concerns while providing a valuable resource for extracting flavonoids for the pharmaceutical industry (6). Hesperidin, a predominant flavonoid in orange peels, possesses antioxidative, antimicrobial, anti-inflammatory, and anti-cancer properties (7), demonstrating potential in SARS-CoV-2 treatment (8). Hesperidin has proven effective against various cancers, including liver, breast, and lung cancers (9). Recent literature indicates that flavonoids like hesperidin and rutin exhibit stronger binding affinity to COVID-19’s main protease than nelfinavir (8, 10), making them promising starting points for COVID-19 therapeutic development. As orange extract may possess antioxidative and antimicrobial attributes, it could be used as an alternative food preservative to extend the shelf life of perishable foodstuffs such as sausages.

Sausages, which possess unique flavours and special tenderness, have been regarded as popular processing foodstuffs among the world’s meat consumers. The casings employed for enclosing minced meats should exhibit two qualities: the strength to endure pressure during stuffing or cooking and the capability to maintain their tender texture (11, 12). Sausage producers encounter difficulties when casings rupture, causing substantial food wastage and impeding efficient sausage manufacturing. In response to this challenge, Feng et al. (13) investigated casing modification using different concentrations of surfactant solutions and storage in slush salt. The modified casings demonstrated an interior structure that was observed to be more permeable, consequently decreasing the occurrence of bursting (13). Nevertheless, sausages are perishable because the sausage filling is made from ground or chopped meat, allowing microorganisms to potentially spread throughout the entire sausage matrix. Previous studies have addressed the addition of orange peels as an alternative to flour or fibre to improve the texture of sausage (14, 15). However, few studies have explored the antimicrobial effects of orange peel extracts in sausage casing modification solutions during long-term storage lasting 1 year, which merits attention.

Adenosine triphosphate (ATP) is prevalent in all living organisms, including various food items where it exists as non-microbial ATP (16). Although it is commonly used for evaluating fish freshness, the ATP content also functions as an indicator of potential microbial contamination since spoilage bacteria influence the transformation of hypoxanthine ribonucleoside into hypoxanthine (Hx). According to Oshita et al. (17), there was a linear relationship between the amount of ATP and plate count, with a determination coefficient of 0.95. Compared with traditional plate count analysis, which typically requires 48 h of incubation and tedious, time-consuming preparation, ATP detection is rapid and reduces analysis time by up to 99.83%. Feng et al. (18) evaluated ATP in ready-to-eat sausage slices stored at 35°C for up to 5 days, with the final ATP contents increased from-5.09 ± 0.01 (day 1) to-5.04 ± 0.09 log (mol/L) (day 5) in the validation group. The fluorescent signal of ATP was identified through the application of two-dimensional Savitzky–Golay second-order differentiation of excitation-emission matrix spectroscopy data acquired from the surface of pork meat, with detection wavelengths at excitation (Ex) = 286 nm and emission (Em) = 386 nm and 412 nm (19). Although ATP measurement offers advantages over sophisticated microbiological methods as mentioned above, there are limitations to measuring ATP: it can only provide the average ATP content of the foodstuff, making it impossible to assess every spot within the measured sample.

Hyperspectral imaging, a technique that associates spectroscopy with imaging to capture spectral and spatial information simultaneously from a subject (20), has emerged as an appealing and rapid method for various foodstuffs such as fruits, vegetables, raw meat, and processed meat products. One primary advantage of HSI technology, compared to other traditional spectroscopic techniques, is its ability to generate pixel-wise prediction maps (also called distribution maps) that illustrate the quantity and distribution of different attributes across samples (21). This image analysis enables precise observation of potential heterogeneity in sausages affected by different casing treatments, which cannot be detected with conventional spectroscopic equipment. The total volatile basic nitrogen (TVB-N) values of cooked beef samples for storing up to 19 days at 4°C have been investigated by HSI, with R_p_^2^ of 0.9401 using the partial least squares regression (PLSR) model and R_p_^2^ of 0.9579 using the least squares-support vector machine (LS-SVM) model (22). The minced beef, chicken, and mutton have been classified by the K-nearest neighbour (KNN), the support vector machine (SVM), and the convolutional neural network (CNN) (23). The authors stated that the deep learning model’s extraction of non-linear features enabled a robust classification of various types of meat using only the myoglobin (Mb) pigment region, surpassing the performance of traditional machine learning algorithms. The suggested framework outperforms conventional spectral methods by achieving an accuracy of 94.00%, surpassing the results obtained through correlation methods. Despite numerous studies on applying plant extracts to processed meat products (24–26), the response of ATP to sausages stuffed in modified casing incorporated with orange extracts after 1 year of storage detected by HSI remains unexplored.

Consequently, the objective of this study is to establish a quantitative relationship between spectral data and the reference ATP content of sausage stuffed in modified casing treated with different concentrations of orange extracts after 1 year of storage, utilising partial least squares regression (PLSR). Subsequently, pixel-wise distribution maps were developed via processing algorithms to depict the ATP content.

## 2 Materials and methods

### 2.1 Ultrasound-assisted extraction (UAE) of waste orange peels

The peels from Valencia sweet oranges (*Citrus sinensis*) were subjected to drying in an oven at 40°C for a duration of 7 days. Subsequently, the dried orange peels were milled using a blade. An average of 10.05 ± 0.08 g of orange powder was then mixed with 200 mL of 100% methanol and immersed in an ultrasonic bath (MCD-10P, ASONE Corporation, Osaka, Japan) with internal dimensions measuring 30.0 cm × 24.0 cm × 15.0 cm and a capacity of 10 L for 85 min. Based on the previous study (27), the optimal temperature of the solvent and the ultrasonic frequency were chosen at 55°C and 40 kHz, respectively. Following this, the sample was centrifugated at 4000 rpm for 15 min at a temperature of 20°C. The resulting supernatant was obtained, and a rotary vacuum evaporator (EYELA NVC-2100, Rikakikai Co. Ltd., Tokyo, Japan) was employed to remove solvent at 45°C under a pressure of 61 hPa for a duration of 8 min. The extracts were then left overnight to ensure complete solvent evaporation. The distilled water was then added to twice the weight of the extract and the sediments were vacuum filtered and stored in a desiccator for at least 7 days at a room temperature of 20°C.

### 2.2 Sausage casing modification and pork sausage production

A length of 30 cm segment of natural hog casing was put into a surfactant solution composed of soy lecithin and soy oil and agitated using a magnetic stirrer set at 500 rpm at ambient temperature for a designated treatment period. Subsequently, the casing segment, without rinsing, was mixed with a slush salt added with lactic acid (LA) for an identical treatment duration. A central composite design was utilised to establish the concentrations of the casing modification solution, the inclusion of orange extracts, the incorporation of LA, and the duration of the treatment. Thirty-two group combinations, with centre points in sextuplicate, were conducted and subsequent data analysis was analysed using Minitab software (version 21.1, Kozo Keikaku Engineering Inc., Tokyo, Japan).

The lean pork meat and back fat (7:3) were cut into small pieces under sterile conditions and meticulously blended with spices and wine. This blended mixture was subsequently filled into modified casings, alongside untreated natural hog casings (as a control), using a stuffing machine (STX-4000-TB2-PD-BL, Electric Meat Grinder & Sausage Stuffer, STX International, Lincoln, NE, USA). The filled sausage was sectioned by twisting and immediately put in a sanitised oven at 45°C drying for 24 h, followed by an aging period at 20°C for an extra 48 h. Subsequently, the dry-cured sausage segments were sectioned within a sterile clean bench, vacuum-sealed, and then stored in a refrigerator at 4°C for a period of 1 year for subsequent analysis. The detailed sausage filling recipe and sausage production can be found in the study of Feng et al. (20).

### 2.3 Hyperspectral imaging system

After storing the sausage sample (both its front and back sides) in a cold room for a year, it was positioned perpendicular to a hyperspectral camera (NH-4-KIT, EBA Japan, Tokyo, Japan). Images were captured using a push-broom line scanning and 151 continuous spectra were obtained with an exposure time of 12.47 ms. To ensure consistent lighting and minimise shadows, a white sheet was employed, complemented by three halogen lamps strategically placed around it. Ice bags were positioned beneath a sterilised black sheet, and two fans were arranged beside the halogen lamps to mitigate temperature rise from the lamp heat. The black sheet was instrumental in creating a distinct separation between the sample and the background. Measurement of each sample was completed within 3 min, including imaging capture, and putting samples back into the packages stored in the fridge, to minimise temperature fluctuation and microbial contamination before following ATP measurement.

The HSAnalyzer software (EBA Japan, Tokyo, Japan) was employed to calibrate the hyperspectral images according to the Equation 1 below:


(1) $$Rci=\frac{r_{1}-r_{2}}{r_{3}-r_{2}}$$

where r_1_ represents the original reflectance image, r_2,_ and r_3_ depict the reflectance images of dark (taken by covering the camera lens in a dark environment) and white (derived from a 100% white reference), respectively. A thresholding-based segmentation criterion was applied to identify the sausage samples in the images. Thus, an initial segmentation mask was created, and all pixels whose reflectance at 695 nm was greater than 0.075 were selected. In addition, to increase the number of samples in the creation of the models, the mean spectrum of each sausage was not simply measured, but the images were subdivided into 15 segments as cross-sections of the same area. From each of these segments, the mean reflectance spectrum was extracted. A reconstruction of the red, green, blue images (RGB images) was also performed from the bands belonging to 600, 550, and 450 nm of hypercubes. These images will be useful for the visualisation of the samples and for the establishment of the distribution maps. All these digital image processes were carried out with MATLAB (R2023b; MathWorks Inc., Natick, MA, United States).

### 2.4 ATP bioluminescence determination

Five grams of the sausage sample after HSI measurement were aseptically measured and mixed with 45 mL of phosphate buffer solution for 2 min using a laboratory stomacher (E-Mix Primo, ASONE Interscience Co., Inc., Osaka, Japan). The sample solution was subsequently diluted in a tenfold series using a phosphate buffer solution. A 0.1 mL portion of the suitable diluted sample solution was injected into a new cuvette placed a luminometer (Luminescenser MCA; Atto Corp., Tokyo), to which 0.1 mL of the extractant (LL-100-2; Toyo B-Net Co., Tokyo) was added. After a 10-s extraction, 0.1 mL of the luciferin-luciferase complex (LL-100-1, Toyo B-Net Co., Tokyo) was mixed with the previous mixtures, the relative light unit (RLU) was measured. ATP levels were determined by calculating the RLU values using a standard curve established with an ATP standard solution (LL-100-1; Toyo B-Net Co., Tokyo) ranging from 10^−13^ to 10^−7^ mol/mL. The experiments were conducted in quadruplicates.

### 2.5 Development and assessment of regression model

Various pre-processing for the spectra, including normalisation, standard normal variate (SNV), multiplicative scatter correction (MSC), first derivative, and second derivative, were executed before the multivariate analysis.

Partial least square regression (PLSR), as an effective multivariate data analysis tool, is widely utilised for datasets with numerous and highly correlated variables in both the independent (X) and dependent (Y) variables (18). In this study, PLSR models were used to establish a relationship between the spectra of the samples and their respective ATP content values. Kennard-stone algorithm was employed for sample selection (28), with a two-thirds fraction allocated for calibration and a one-third fraction for prediction.

Model accuracy was gauged through metrics such as the determination coefficient in calibration (R_c_^2^), prediction (R_p_^2^), and full cross-validation (R_cv_^2^), as well as the root mean square error of calibration (RMSEC), prediction (RMSEP), and full cross-validation (RMSECV), along with the absence of a significant lack of fit. Typically, the robustness of the PLSR model exhibits a high R^2^ coupled with a low RMSE, alongside minimal discrepancies between RMSEC and RMSECV (29). The development of models as well as the calculation of goodness of fits were carried out with the statistics and machine learning toolbox included in MATLAB (R2023b; MathWorks Inc., Natick, MA, United States).

### 2.6 Establishment of ATP distribution map

The ATP distribution maps of sausage using various modified casings were generated based on calibration models incorporating the full spectrum. Since HSI captures extensive spatial and spectral details in a 3D matrix, it needs to be transformed into a 2D matrix. This transformation ensures that each row represents the spectrum of a pixel, while columns signify the wavelengths. Multiplying this matrix by its respective regression coefficient allows the vector to be restructured, producing a 2D colour image. This approach effectively showcases the colour variations in sausages using a linear colour scale, presenting a distribution map of colour differences. All computations were performed using MATLAB software (R2023b; MathWorks Inc., Natick, MA, United States).

## 3 Results and discussion

### 3.1 Concurrent impact on the ATP content of sausages stuffed in various casings

Table 1 displays the linear, quadratic, and interaction of ATP content of sausage response to each variable. The quadratic polynomial regression equation predicting ATP content based on soy lecithin addition (X_α_), soy oil addition (X_β_), treatment duration (X_γ_), addition of lactic acid (X_δ_), and addition of orange extracts (X_ε_) in uncoded units is presented below:


(2) $$\begin{array}{l} Y_{ATP}=-50.4+2.15\;X_{\alpha }+7.18\;X_{\beta }+0.53\;X_{\gamma }+0.98\;X_{\delta }+\\ 44.5\;X_{\varepsilon }-0.022\;X_{\alpha }^{2}-1.194\;X_{\beta }^{2}-\\ 0.00182\;X_{\gamma }^{2}+0.013\;X_{\delta }^{2}-23.8\;X_{\varepsilon }^{2}-\\ 0.082\;X_{\alpha }X_{\beta }-0.0247\;X_{\alpha }X_{\gamma }-\\ 0.065\;X_{\alpha }X_{\delta }+6.21\;X_{\alpha }X_{\varepsilon }-\\ 0.0167\;X_{\beta }X_{\gamma }+0.006\;X_{\beta }X_{\delta }-\\ 5.45\;X_{\beta }X_{\varepsilon }-0.0091\;X_{\gamma }X_{\delta }+\\ 0.064\;X_{\gamma }X_{\varepsilon }-2.51\;X_{\delta }X_{\varepsilon } \end{array}$$

**Table 1:** Regression coefficients and analysis of variance for the ATP regression models.

Coded coefficients				Analysis of variance			
Term	Coefficients	SE coefffients	*p*-value	Df	Seq SS	Contribution	Adjust sum of squares
Constant	−5.539	0.54	0.000	5	6.9374	10.07%	6.9374
X_α_	0.406	0.276	0.170	1	3.9465	5.73%	3.9465
X_β_	−0.081	0.276	0.773	1	0.1593	0.23%	0.1593
X_**γ**_	−0.249	0.276	0.387	1	1.4857	2.16%	1.4857
X_δ_	−0.054	0.276	0.849	1	0.0696	0.10%	0.0696
X_**ε**_	−0.231	0.276	0.421	1	1.2764	1.85%	1.2764
$X_{\alpha }^{2}$	−0.027	0.250	0.916	1	0.1065	0.15%	0.0211
$X_{\beta }^{2}$	−0.466	0.250	0.089	1	4.9531	7.19%	6.3834
$X_{\gamma }^{2}$	−0.410	0.250	0.129	1	4.2330	6.14%	4.9246
$X_{\delta }^{2}$	0.030	0.250	0.906	1	0.1360	0.20%	0.0270
$X_{\varepsilon }^{2}$	−0.450	0.250	0.099	1	5.9456	8.63%	5.9456
X_α_X_β_	−0.057	0.338	0.870	1	0.0517	0.08%	0.0517
X_α_X_γ_	−0.411	0.338	0.250	1	2.7000	3.92%	2.7000
X_α_X_δ_	−0.108	0.338	0.755	1	0.1873	0.27%	0.1873
X_α_X_ε_	0.948	0.338	0.017	1	14.3758	20.87%	14.3758
X_β_X_γ_	−0.156	0.338	0.653	1	0.3906	0.57%	0.3906
X_β_X_δ_	0.006	0.338	0.986	1	0.0006	0.00%	0.0006
X_β_X_ε_	−0.468	0.338	0.193	1	3.5109	5.10%	3.5109
X_γ_X_δ_	−0.204	0.338	0.559	1	0.6660	0.97%	0.6660
X_γ_X_ε_	0.132	0.338	0.704	1	0.2778	0.40%	0.2778
X_δ_X_ε_	−0.517	0.338	0.154	1	4.2819	6.22%	4.2819
Error				11	20.1332	29.23%	20.1332
Lack-of-fit				6	13.7489	19.96%	13.7489
Pure Error				5	6.3843	9.27%	6.3843
Total				31	68.8873	100.00%	

Xα, soy lecithin addition, Xβ, soy oil addition, Xγ, treatment duration, Xδ, lactic acid, Xε, orange extracts. Df, degree of freedom; SE coefficients, standard error of the regression coefficients; Seq SS, sequential sum of squares.

**Figure 1:**
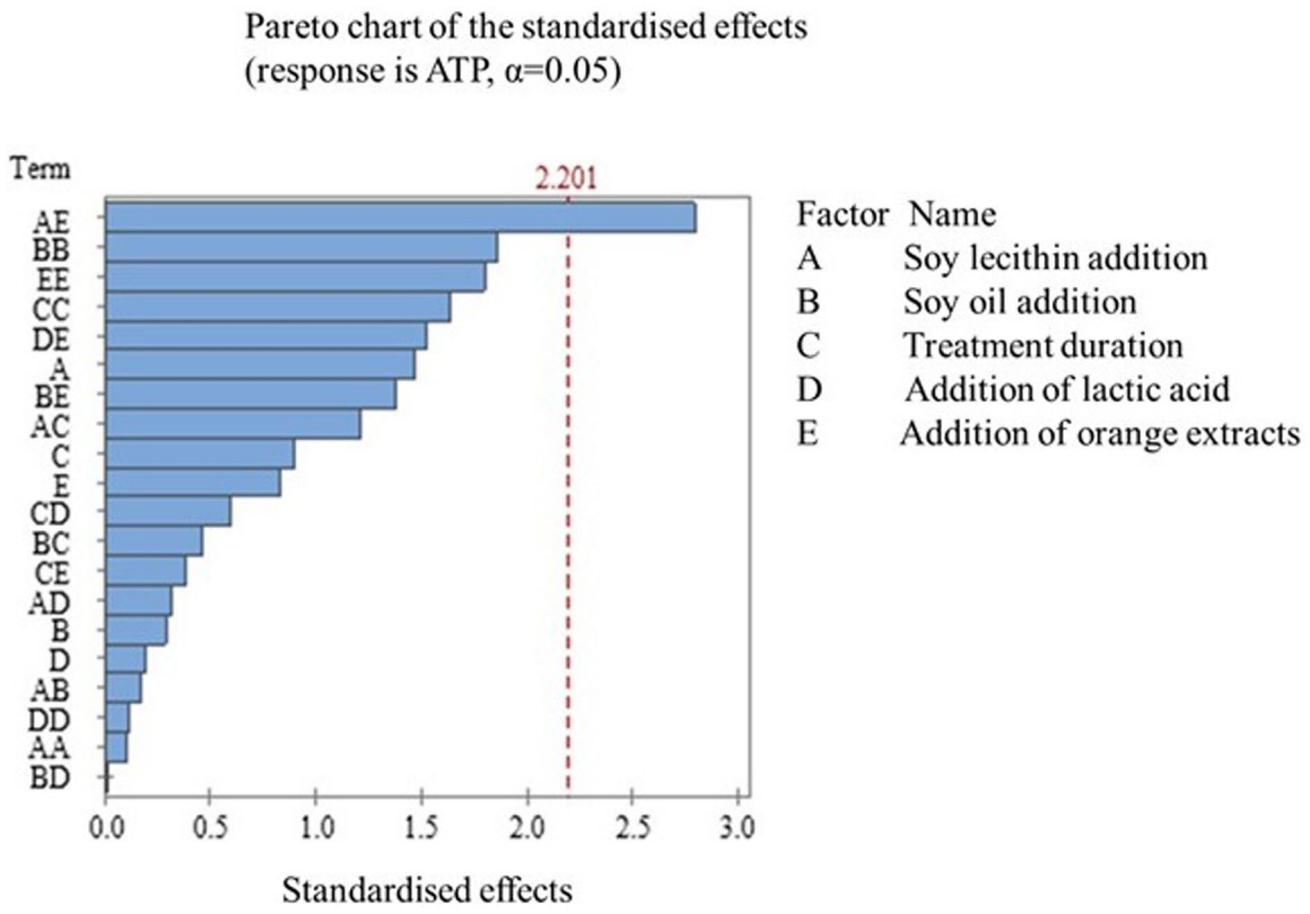
Pareto chart of the standardised effects.

**Figure 2:**
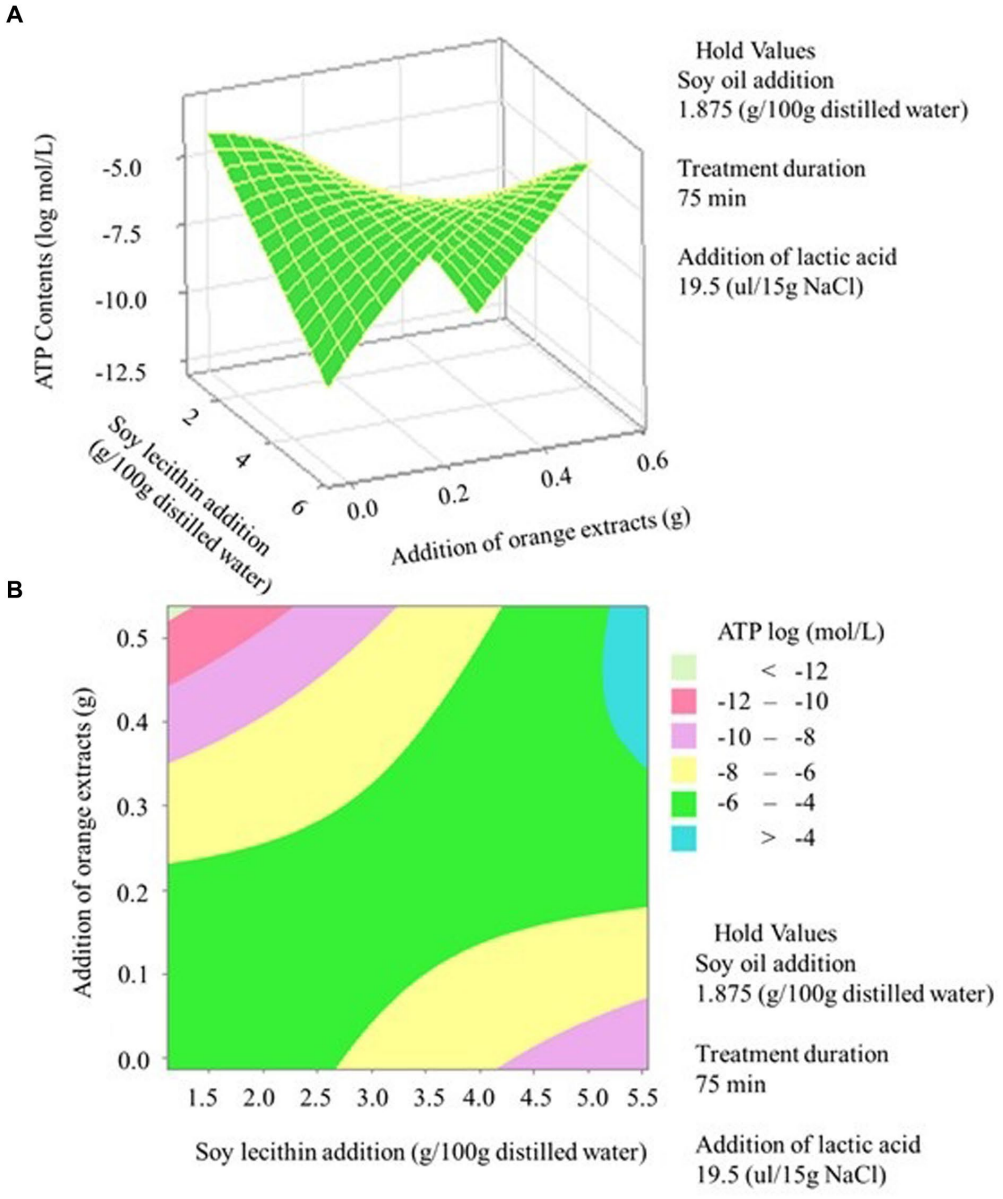
ATP contents of the sausage encased in modified casings as influenced by the addition of orange extracts and soy lecithin concentration. **(A)** three-dimensional surface plot; **(B)** two-dimensional contour plot.

The established regression model (Equation 2) for ATP possessed an R^2^ value of 70.77%, with a statistically insignificant lack of fit (*p > 0.05*) (Table 1). Figure 1 illustrates that the interactive effects of soy lecithin and orange extracts addition on ATP content were observed at a 5% significant level. Response surface methodology (RSM) is a robust statistical approach known for optimizing intricate processes (30, 31) and offering insightful experimental designs, such as the central composite design. This method is crucial for understanding the correlation between process parameters and the quality characteristics of food products (32), particularly in situations where the influence of variables on the outcome is uncertain. The measured and predicted ATP values can be observed in Table 2. ATP value got higher than-8 log mol/L when a lower orange extract addition (<0.2%) was combined with soy lecithin regardless of its concentration (Figure 2). It is well-known that a higher ATP value implies a higher microbial activity (17, 18). As stated by Oshita et al. (17), a linear correlation was observed between the quantity of ATP and plate count, yielding a determination coefficient of 0.95. This finding also aligns with the current observation, where the total plate counts for treatments No. 7, 16, and 28 were 6.92 ± 0.71 Log (cfu/g), 7.52 ± 0.69 Log (cfu/g), and 8.06 ± 0.89 Log (cfu/g), respectively. Accordingly, the average ATP contents for treatments No. 7, 16, and 28 were-8.66 ± 0.00 log (mol/L), −6.14 ± 0.01 log (mol/L), and − 4.37 ± 0.14 log (mol/L), respectively. A lower ATP value was achieved when a higher orange extract addition was associated with a lower soy lecithin concentration, which indicates the antimicrobial attribute of the orange extracts. The antimicrobial properties of the Thomson navel orange extracts that were soxhlet-extracted by hexane (non-polar extract) and methanol (polar extract) have been studied (33). For gram-positive bacteria like *Listeria monocytogenes*, the inhibitory halo diameter of the polar extract was observed to be significantly smaller (9.66 ± 0.06 mm) than that of the non-polar extract (10.33 ± 0.03 mm) (*p < 0.05*). However, the orange extracts indeed showed antibacterial activity irrespective of the extraction method. While vacuum packaging reduces oxygen availability, which many spoilage bacteria require, it can create an anaerobic environment that may enable the growth of certain pathogens such as *Listeria monocytogenes*. Additionally, this pathogenic bacterium can survive and even survive at refrigeration temperatures. It is a concern in ready-to-eat foods like vacuum-packaged sausages, as it can cause listeriosis, a serious foodborne illness. The addition of orange extracts may prevent the vacuum-packaged sausages from *Listeria monocytogenes.* The study of Chakroun et al. (33) also investigated the antibacterial activity of gram-negative bacteria such as *Escherichia coli.* It was found that the polar extract exhibited a zone of inhibition of 9.33 ± 0.03 mm, followed by 9 ± 0.01 mm and 8 ± 0.01 mm for the non-polar extract and hesperidin, respectively. This result illustrates that orange extracts may have higher antibacterial activity than hesperidin alone. The other antibacterial components included in the orange extracts may contribute to this phenomenon. Besides hesperidin, naringin and other phenolic compounds were observed in orange extracts, which may disrupt the cell walls of bacteria, leading to cell lysis and death. It is reported that phenolic compounds can disrupt the integrity of bacterial cell membranes by interacting with phospholipids and proteins (34). This disruption can lead to leakage of cellular contents and ultimately cell death (35). Furthermore, certain phenolic compounds have been shown to generate reactive oxygen species (ROS) within bacterial cells. ROS can cause oxidative damage to cellular components, including the cell wall, leading to cell death (36). Moreover, some components in orange extracts may inhibit microbial enzymes essential for their survival and replication. By disrupting these enzymes, the growth and proliferation of microorganisms can be hindered.

**Table 2:** Matrix of central composite design for modified casing treatment combinations and the response of measured and predicted ATP values.

Treatment	Soy lecithin addition (X_α_, g/100 g distilled water)	Soy oil addition (X_β_ g /100 g *distilled water*)	Treatment duration (X_γ_, min)	Addition of lactic acid (ul/15 g NaCl, X_δ_)	Addition of orange extracts (X_ε_, g)	Measured ATP value (Y, log mol/L)	Predicted ATP value (log mol/L)
1^*^	3.33	1.875	75	292.5	0.28	−7.54 ± 0.44	−5.54
2	3.33	1.875	75	337.5	0.28	−6.85 ± 0.05	−5.53
3	4.44	1.250	90	270.0	0.42	−4.84 ± 0.13	−4.62
4	3.33	1.875	75	247.5	0.28	−5.49 ± 0.45	−5.31
5	2.22	2.500	90	315.0	0.13	−4.62 ± 0.03	−5.40
6^*^	3.33	1.875	75	292.5	0.28	−4.92 ± 0.00	−5.54
7	2.22	2.500	60	315.0	0.42	−8.66 + 0.00	−9.33
8	1.11	1.875	75	292.5	0.27	−8.19 ± 0.98	−6.46
9	3.33	1.875	105	292.5	0.28	−8.04 ± 0.98	−7.68
10	3.33	1.875	45	292.5	0.28	−7.82 ± 0.48	−6.68
11^*^	3.33	1.875	75	292.5	0.28	−4.85 ± 0.43	−5.54
12	2.22	1.250	90	315.0	0.41	−7.62 ± 0.33	−8.18
13	4.44	2.500	60	315.0	0.13	−4.85 ± 0.06	−5.33
14	4.44	2.500	60	270.0	0.43	−5.30 ± 0.13	−5.19
15	4.44	1.250	60	315.0	0.42	−4.99 ± 0.11	−5.24
16	2.22	1.250	90	270.0	0.13	−6.14 ± 0.01	−6.71
17	3.33	3.125	75	292.5	0.28	−8.17 ± 0.98	−7.57
18	4.44	1.250	90	315.0	0.13	−7.63 ± 0.16	−7.99
19	2.22	1.250	60	315.0	0.13	−4.90 ± 0.17	−5.95
20	4.44	2.500	90	315.0	0.43	−7.92 ± 0.81	−7.91
21	2.22	1.250	60	270.0	0.41	−7.68 ± 0.68	−8.14
22	4.44	1.250	60	270.0	0.13	−7.15 ± 0.17	−7.42
23	3.33	1.875	75	292.5	0.57	−7.95 ± 0.66	−7.80
24	5.55	1.875	75	292.5	0.28	−4.60 ± 0.05	−4.84
25	2.22	2.500	90	270.0	0.42	−7.95 ± 0.50	−8.14
26^*^	3.33	1.875	75	292.5	0.28	−4.90 ± 0.01	−5.54
27	4.44	2.500	90	270.0	0.13	−7.94 ± 0.40	−7.95
28^*^	3.33	1.875	75	292.5	0.28	−4.37 ± 0.14	−5.54
29^*^	3.33	1.875	75	292.5	0.28	−5.15 ± 0.27	−5.54
30	2.22	2.500	60	270.0	0.13	−5.61 ± 0.03	−6.30
31	3.33	1.875	75	292.5	−0.01	−8.23 ± 1.13	−6.88
32	3.33	0.625	75	292.5	0.27	−8.14 ± 0.78	−7.24
Control	–	–	–	–	–	−8.06 ± 0.67	

^*^ Central points.

### 3.2 Spectral overview and calibration models at full wavelengths

**Figure 3:**
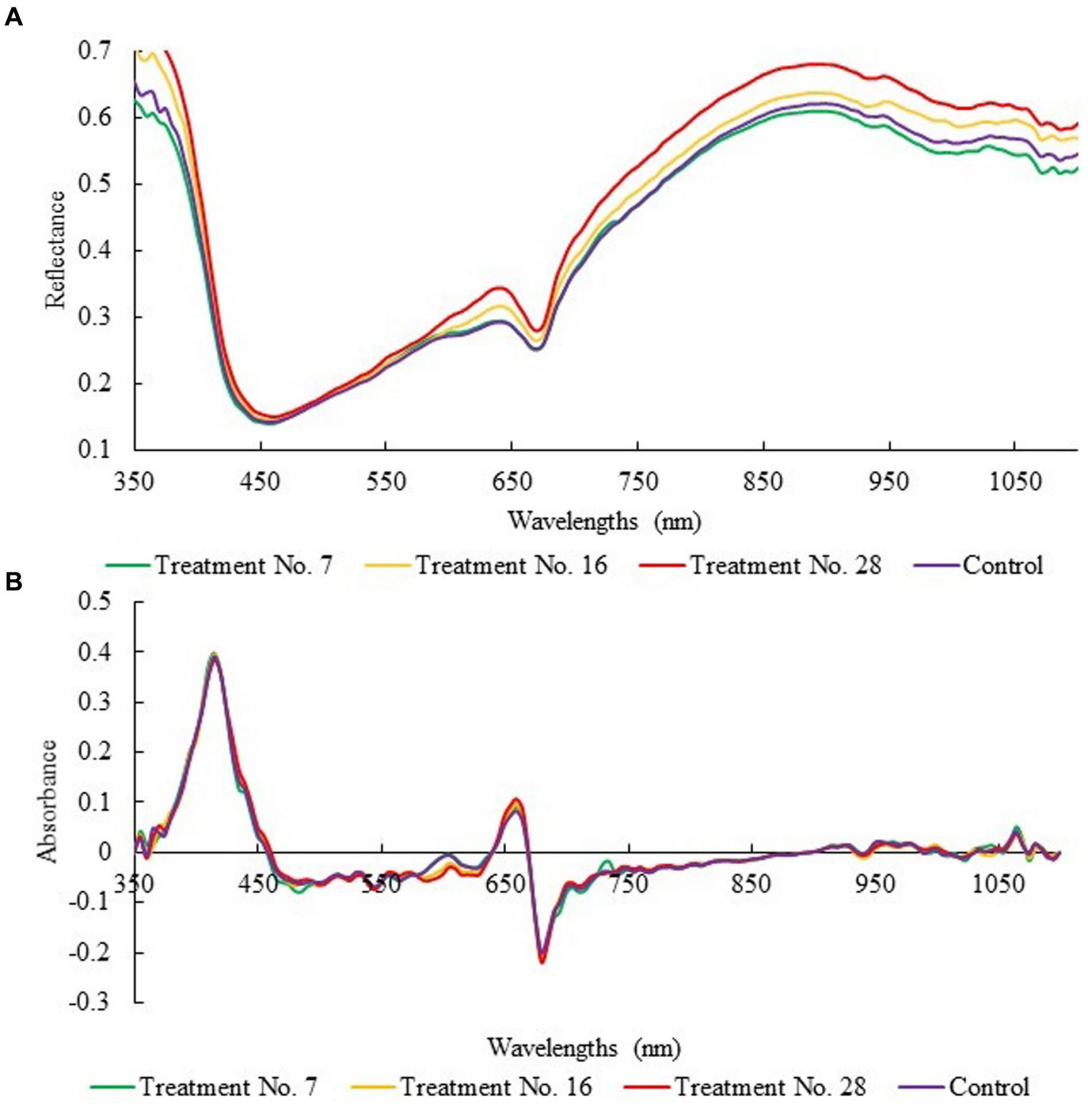
Reflectance **(A)** and absorbance with pre-treatment of standard normal variate followed by 1^st^ derivative **(B)** of the representative sausage stuffed in modified casings with treatments No. 7, 16, 28, and Control.

**Figure 4:**
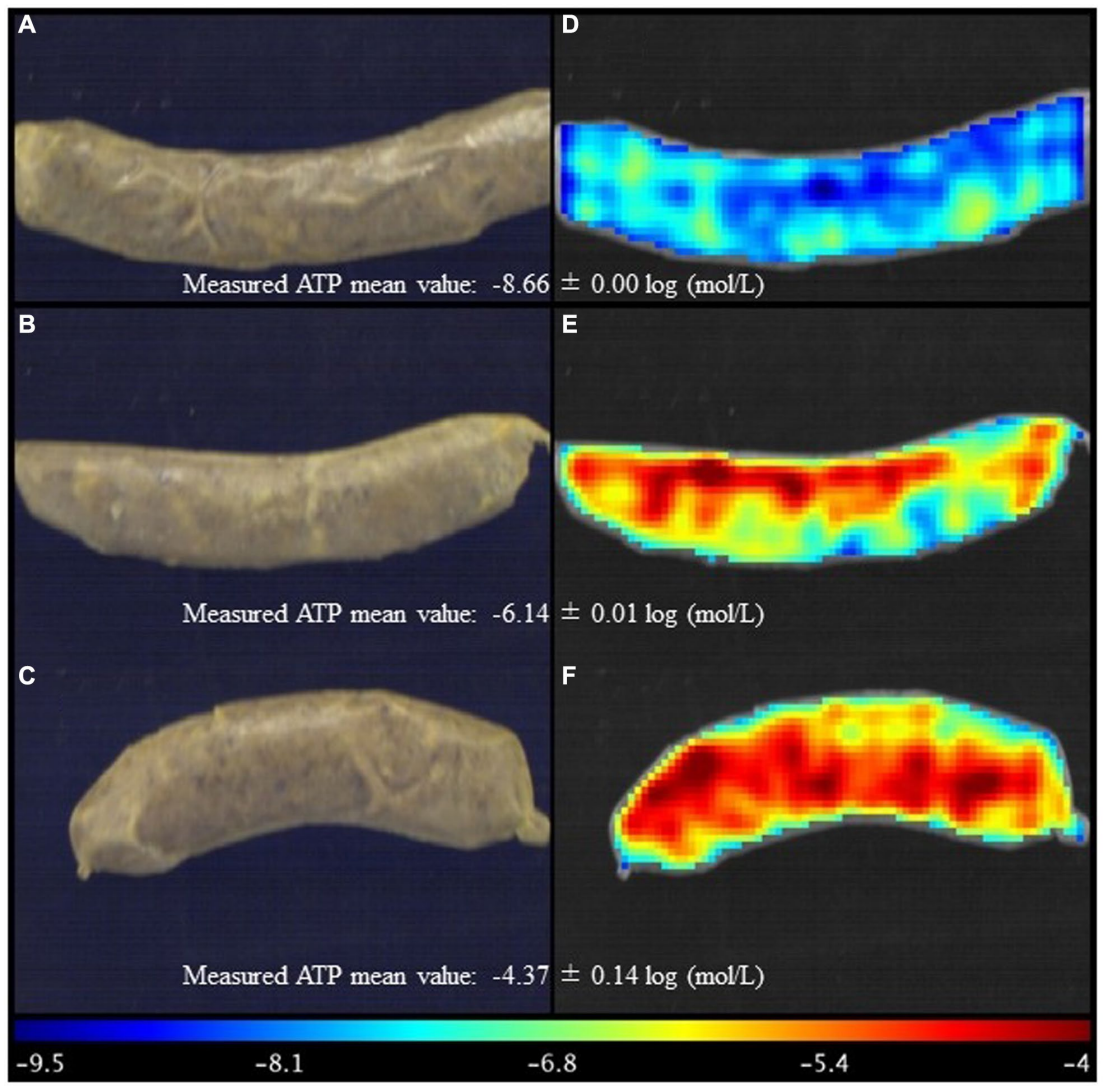
Visualisation of ATP content distribution map for representative sausage stuffed in the modified casing with treatment No. 7 **(A,D)**, treatment No. 16 **(B,E)**, and treatment No. 28 was the central point in the central composite design **(C,F)**.

Figure 3 illustrates the reflectance spectra for representative sausage sampled stuffed in modified casing modified by treatments No. 7, 16, 28, and control. The distinct spectra observed in Figure 3 could be attributed to variations in ATP content. There is a trend where a higher ATP value has a higher reflectance spectrum (Figure 4A). The enhancements in variations were particularly notable in the 350–450 nm and 600–750 nm range (Figure 4B). As the wavelength range between 600 nm and 700 nm is typically associated with oxymyoglobin formation (37), these observations indicate a relationship between ATP levels and meat colour. Motoyama et al. have stated that in instances where few microorganisms are present on the meat surface and the meat colour appears bright red, both microorganisms and myoglobin derivatives can access oxygen. However, as microbial growth occurs, a microbial film impedes myoglobin derivatives from accessing oxygen, resulting in the reduction of metmyoglobin to deoxymyoglobin. Consequently, the associated spectra for metmyoglobin, deoxymyoglobin, and oxymyoglobin are 632, 556, and 580, respectively (38). Table 3 showcases the statistical parameters of PLSR using different pre-processing treatments to evaluate ATP levels within the calibration, prediction, and cross-validation datasets. All these spectral pre-treatments were carried out using the absorbance spectra. The treatment that yielded the best results was the transformation from reflectance to absorbance, followed by SNV and a 1^st^ derivative (R_c_^2^ = 0.7649, R_p_^2^ = 0.6629; RMSEC = 0.7384, RMSEP = 0.8880). This first derivative was applied by measuring the increment between consecutive wavelengths after applying a second-degree Savitzky–Golay smoothing with a five-band window. This smoothing before the spectral derivative was also optimised to minimise spectral noise without negatively affecting the analytical information contained in the spectra. On the other hand, it should be noted that these models were developed from the extraction of fifteen different sections of the sausages in which there were small spectral differences. All sections of the same sausage were assigned the same reference value when constructing the chemometric models. In a comparable study, the R_p_^2^ and RMSEP values for ATP prediction in ready-to-eat sausages using reflectance with SNV and 1^st^ derivative methods were reported as 0.8014 and 0.016, respectively (18). The spectra pre-treatment indeed improves the performance of the model compared with the raw data. This improved accuracy could be due to reduced scattering effects or the removal of background noise. Goodness-of-fit indicators depend on many factors. The utilisation of cross-sections from the samples may contribute to achieving better goodness of fit. In the present work, the goodness-of-fit indicators have decreased slightly. However, it must be taken into account that the images were acquired on complete samples, including the casing, which can negatively affect the measurement. This is why the current R_p_^2^ and RMSEP values are considered adequate.

**Table 3:** Statistical parameters of partial least square regression with raw and pre-processing spectra.

Treatments	Calibration group		Prediction group		Cross-validation	
	R_c_^2^	Root mean square error of calibration (%)	R_p_^2^	Root mean square error of prediction (%)	R_cv_^2^	Root mean square error of cross-validation (%)
Raw data (reflectance)	0.5978	0.9659	0.5211	1.0585	0.5943	0.9701
Raw data (absorbance)	0.6031	0.9595	0.5244	1.0548	0.5943	0.9111
MSC	0.5643	1.0052	0.4878	1.0947	0.6081	0.9546
1^st^ Derivative	0.6458	0.9064	0.5368	1.0410	0.6391	0.9170
SNV	0.5812	0.9856	0.5207	1.0589	0.6312	0.9260
2^nd^ Derivation	0.6180	0.9412	0.5410	1.0362	0.6508	0.9016
Normalisation	0.5948	0.9694	0.5173	1.0627	0.5948	0.9712
SNV + 1^st^ Derivative	0.7649	0.7384	0.6629	0.8880	0.7025	0.8321
1^st^ Derivative + SNV	0.6339	0.9215	0.5434	1.0336	0.6590	0.8905

MSC, multiplicative scatter correction; SNV, standard normal variate; Rc2, coefficients of determination for calibration; Rp2, coefficients of determination for prediction; Rcv2, coefficients of determination for cross-validation. All spectral pre-treatments from MSC downwards were carried out on the absorbance spectra.

### 3.3 ATP distribution map of representative sausage

The main advantage of hyperspectral imaging is to be able to obtain spectral information and its spatial distribution over the samples. Therefore, when regression models are obtained, it is possible to produce distribution maps whose visualisation improves the understanding of the results. To do this, the pixels of the region of interest were located and unfolded in a table and the coordinates of each pixel were stored for later reconstruction. The same spectral pre-treatment was applied to these spectra as in the creation of the models and they were matrix multiplied by the regression vector by adding the independent term. The values obtained corresponded to the ATP in the same units with which the models were created (− log). Finally, the data were refolded according to the original image and a colour scale was used for better visualisation. These distribution maps were generated alongside the reconstructed RGB image to improve the understanding of the images. Observing the RGB images was challenging to differentiate the samples with the naked eye (Figures 4A–C). However, as shown in Figures 4D–F, the ATP content levels and their locations within the sausage are distinctly visible. This offers a swift means to evaluate the optimal casing-modified treatments based on ATP content in sausage, highlighting the primary benefit of HSI technology over traditional spectroscopic approaches (39). The reason lies in the fact that pixels with analogous spectral traits will produce comparable predicted values, resulting in consistent representations on the prediction map. Consequently, the different ATP levels of sausages stuffed in the modified casing can be detected non-invasively through hyperspectral imaging.

The novelty of the current study includes:

1. The elucidation, through response surface methodology, of the concurrent effects of five variables (varying additions of soy lecithin, soy oil, treatment duration, and addition of lactic acid, addition of orange extracts) on the ATP content of sausages has been done.
2. The ATP contents in sausages stuffed in different modified casing treatments along with the addition of orange extracts during long-time storage have been investigated for the first time. Prediction maps vividly illustrate the ATP content variations at each pixel in response to different casing treatments. These findings enhance the understanding of the interaction between ATP content and orange extracts, offering valuable insights for future investigations.
3. The relationship between ATP content and spectra obtained from the surface of cylindrical sausages with modified casings was established using PLSR for the first time.

The ability of hyperspectral imaging to non-destructively estimate ATP content can be leveraged for assessing the shelf life of perishable food items. By monitoring changes in ATP levels over time, it may be possible to predict and manage food spoilage more effectively. HSI can be utilised to detect early signs of food spoilage based on changes in ATP distribution. By identifying regions with elevated ATP concentrations, manufacturers can take corrective actions to prevent further spoilage and minimise product waste. In addition, ATP distribution mapping can be integrated into traceability systems to track the origin and processing history of food products throughout the supply chain. By monitoring ATP content at various stages of production and distribution, stakeholders can ensure compliance with quality standards and regulatory requirements. Further research into hyperspectral imaging for ATP content estimation can lead to the development of advanced analytical techniques and algorithms. This may enable more accurate and reliable measurements, expanding the scope of applications in the food industry and related fields. The integration of hyperspectral imaging with ATP content estimation holds promise for enhancing various aspects of food production, processing, and safety, as well as contributing to advancements in agricultural practices and research.

## 4 Conclusion

This research tackled the challenges associated with the implementation and utilisation of a hyperspectral imaging system to estimate adenosine triphosphate content in sausages stuffed in different modified casing treatments. Various pre-treatment combinations were applied to raw reflectance spectra, revealing that SNV + 1^st^ derivative absorbance spectra exhibited superior performance compared to other pre-processed spectra treatments. The PLSR model, developed using spectra pre-treated with the SNV followed by the 1^st^ derivative, yielded satisfactory results with an R_p_^2^ of 0.6629 and RMSEP of 0.8880. For the first time, a distribution map generated by HSI associated with MATLAB visualised the changes in ATP content in sausages in response to different casing treatments. Hyperspectral imaging emerges as a promising tool for the rapid and non-destructive measurement of ATP content, enabling both quantification and visualisation of ATP evolution in sausages during long-term storage.

## Data availability statement

The original contributions presented in the study are included in the article/supplementary material, further inquiries can be directed to the corresponding author.

## Author contributions

C-HF: Conceptualization, Data curation, Formal analysis, Funding acquisition, Investigation, Methodology, Project administration, Resources, Software, Validation, Visualisation, Writing – original draft, Writing – review & editing. HA: Conceptualization, Funding acquisition, Project administration, Resources, Supervision, Writing – review & editing. FJR-P: Data curation, Formal analysis, Validation, Visualisation, Writing – review & editing.
